# Bringing Rehabilitation Home: A Policy and Practice Perspective on COPD Management

**DOI:** 10.5041/RMMJ.10567

**Published:** 2026-01-28

**Authors:** Abins Thozhuthinkal Kasim, Ravi Gaur, Nitesh Manohar Gonnade, Nagma Sheenam

**Affiliations:** Department of Physical Medicine and Rehabilitation, All India Institute of Medical Sciences, Jodhpur, Rajasthan, India

**Keywords:** Balance training, chronic obstructive pulmonary disease, cognitive training, home-based pulmonary rehabilitation, multidimensional rehabilitation, tele-rehabilitation

## To the Editor

We thank Thakur and Bhatia for their insightful letter, “Home-based Pulmonary Rehabilitation in COPD: Bridging Evidence and Practice for Comprehensive Patient-centered Care,”^1^ and for their gracious recognition of our study, “From Breathlessness to Better Living: Transforming COPD Care with Home-based Pulmonary Rehabilitation.”^2^ We appreciate their remarks, which underscore the value of our findings and enrich the discussion with constructive suggestions to further strengthen home-based pulmonary rehabilitation (PR). We share their vision for a more accessible, patient-centered, and holistic approach to chronic obstructive pulmonary disease (COPD) care and welcome this opportunity to continue the dialogue.

Thakur and Bhatia aptly highlighted the key outcomes of our 12-week home-based PR program, which we are pleased to reaffirm. Participants demonstrated significant improvements in lung function—particularly in forced vital capacity (FVC) and forced expiratory volume in 1 second (FEV_1_) (both *P*<0.001)—along with marked reductions in disability as evidenced by lower World Health Organization Disability Assessment Schedule 2.0 (WHODAS 2.0) scores. Gains were most notable in the “life activities” and “participation” domains (*P*<0.001), reflecting improved daily functioning and social engagement. These findings confirm that structured home-based PR can deliver meaningful physiological and functional benefits.

We appreciate the authors’ acknowledgment of our work’s broader implications. As they observed, our study reinforces PR as a holistic, patient-centered approach to COPD—complementing or even substituting pharmacologic therapy. Improvements in FVC, FEV_1_, and functional and social outcomes together illustrate PR’s core impact: translating better breathing into better living.

Thakur and Bhatia have appropriately highlighted certain limitations of our study, and we concur with their insights. The lack of a control group restricts causal interpretation, as observed improvements may partly reflect natural recovery, participant motivation, or a Hawthorne effect. Our study was designed as an initial feasibility assessment, and we agree that a randomized controlled trial would provide more definitive evidence. In line with their recommendation, we are planning a controlled study to validate the efficacy of home-based PR more robustly.

We also acknowledge their point regarding adherence measurement. Our reliance on self-reported data from monthly phone calls, while practical, may have overestimated actual adherence. We appreciate their suggestion to adopt tele-rehabilitation tools—such as wearable sensors, mobile applications, and video sessions—for objective monitoring and real-time feedback. Incorporating these technologies can improve both accuracy and patient engagement, offering a more reliable and motivating framework for future studies to assess and enhance exercise compliance.

We appreciate the authors’ thoughtful observations on our outcome measures. Although participants showed meaningful improvements in several disability domains, cognitive and mobility gains remained limited. As noted, COPD-related cognitive decline from chronic hypoxemia and inflammation can hinder self-management, while mobility deficits often stem from weakness, balance loss, and sarcopenia—areas not fully addressed by endurance training alone. We endorse their constructive suggestion to broaden rehabilitation components to target these domains more directly. Future home-based PR models could incorporate cognitive-behavioral and task-oriented elements—such as goal setting, education, attentional control, and reframing of activity-related anxiety—to enhance cognitive engagement during exercise. Emerging evidence indicates that physical activity supports attention, memory, and executive function in older adults with COPD; integrating structured cognitive stimulation may further mitigate cognitive decline and strengthen adherence and self-management.^3^,^4^

Incorporating multimodal strategies can further enhance the scope and impact of home-based PR. Resistive strength and balance training—through band- or weight-based exercises and gait stabilization drills—can address lower-limb weakness and postural instability that endurance training alone may not correct. Evidence from large multi-center trials supports that adding structured balance components to standard PR improves lower-limb strength and immediate balance, though sustained fall reduction remains variable. Telehealth and digital adherence tools, including wearable sensors, smartphone applications, and video check-ins, can enable real-time feedback, ensure correct exercise performance, and sustain motivation between sessions. Finally, a multidimensional support framework encompassing nutritional counseling, psychological care, and family or peer-based social reinforcement can help address the broader physical, emotional, and social determinants of disability—ensuring that rehabilitation treats not just symptoms, but the whole patient.^5^–^7^

We believe that integrating these components will help realize the letter writers’ call for truly multidimensional rehabilitation. Although our original program emphasized endurance training, breathing exercises, and education, future versions will expand to include cognitive and dual-task activities, progressive strength and balance exercises, and telehealth-based supervision for remote guidance and motivation. These additions aim to enhance mobility and cognitive function, while also improving adherence, engagement, and overall patient satisfaction.

One of the most compelling points raised by the authors is the relevance of home-based PR in resource-limited, high-burden settings like India. We fully share their view that delivering rehabilitation at home can greatly enhance accessibility and scalability. Conventional center-based programs are often constrained by distance, cost, and limited availability, leaving many COPD patients underserved. Our intent in developing a home-based model was precisely to address these barriers, and we appreciate the authors for emphasizing its importance within under-resourced healthcare systems.^8^

We concur with the authors that delivering home-based PR through trained physiotherapists, nurses, or community health workers is a practical and cost-effective way to expand access. In our study, family involvement served as an important support system, and we agree that structured caregiver participation could further enhance adherence and motivation. We also share their view that by reducing exacerbations and hospitalizations, home-based PR can lessen healthcare utilization and economic burden—an especially relevant benefit for low- and middle-income settings.

We fully support their recommendation to integrate home-based PR into national respiratory-health programs and to include it under public or private insurance coverage. Policy-level advocacy will be vital for scaling and sustaining such efforts. Likewise, training healthcare providers is essential to ensure standardized, high-quality program delivery. Future research should also address equity considerations, examining how gender, socioeconomic status, or rural–urban differences affect access and outcomes. Our shared vision is to make PR a universally accessible standard of care, not a privilege limited by geography or resources.

Thakur and Bhatia’s comments emphasize the shared objective of advancing COPD rehabilitation through patient-centered, home-based models. The response outlines plans to build on these insights by incorporating long-term follow-up, telehealth integration, and individualized care pathways. The broader vision focuses on refining PR into a more accessible, evidence-informed framework that promotes functional recovery and improved quality of life for people living with COPD.

## Abbreviations

COPD: chronic obstructive pulmonary disease
FEV1: forced expiratory volume in 1 second
FVC: forced vital capacity
PR: pulmonary rehabilitation
WHODAS 2.0: World Health Organization Disability Assessment Schedule 2.0.
